# Relationship between the expression of ARHGAP25 and RhoA in non-small cell lung cancer and vasculogenic mimicry

**DOI:** 10.1186/s12890-022-02179-5

**Published:** 2022-10-07

**Authors:** Fan Shi, Jiatao Wu, Qianhao Jia, Kairui Li, Wenjuan Li, Yuqi Shi, Yufei Wang, Shiwu Wu

**Affiliations:** 1 Department of Pathology, The First Affiliated Hospital of Bengbu Medical College, Changhuai road 287, Bengbu, 233000 Anhui People’s Republic of China; 2 Department of Pathology, Bengbu Medical College, 2600 Donghai Avenue, Bengbu, Anhui China

**Keywords:** ARHGAP25, RhoA, Rho GTPase, Vasculogenic mimicry, NSCLC, Prognosis

## Abstract

**Background:**

Vasculogenic mimicry (VM) is a recently identified pattern of blood supply to tumor tissue. It has long been considered a functional element in the metastasis and prognosis of malignant tumors. Both Rho GTPase-activating protein 25 (ARHGAP25) and Ras homolog family member A (RhoA) are effective predictors of tumor metastasis. In this study, we examined the expression levels of ARHGAP25 and RhoA and the structure of VM in non-small cell lung cancer (NSCLC). At the same time, we used cytology-related experiments to explore the effect of ARHGAP25 on the migration ability of tumor cells. Furthermore, we analyzed the interaction between the three factors and their association with clinicopathological characteristics and the five-year survival time in patients using statistical tools.

**Methods:**

A total of 130 well-preserved NSCLC and associated paracancerous tumor-free tissues were obtained. Cell colony formation, wound healing, and cytoskeleton staining assays were used to analyze the effect of ARHGAP25 on the proliferation and migration ability of NSCLC cells. Immunohistochemical staining was used to determine the positivity rates of ARHGAP25, RhoA, and VM. Statistical software was used to examine the relationships between the three factors and clinical case characteristics, overall survival, and disease-free survival.

**Results:**

Cell colony formation, wound healing, and cytoskeleton staining assays confirmed that ARHGAP25 expression affects the proliferation and migratory abilities of NSCLC cells. ARHGAP25 positivity rates in NSCLC and paracancerous tumor-free tissues were 48.5% and 63.1%, respectively, whereas RhoA positivity rates were 62.3% and 18.5%, respectively. ARHGAP25 had a negative relationship with RhoA and VM, whereas RhoA and VM had a positive relationship (*P* < 0.05). ARHGAP25, RhoA, and VM affected the prognosis of patients with NSCLC (*P* < 0.05) according to Kaplan–Meier of survival time and Cox regression analyses. Furthermore, lowering ARHGAP25 expression increased NSCLC cell proliferation and migration.

**Conclusions:**

ARHGAP25 and RhoA expression is associated with VM and may be of potential value in predicting tumor metastasis, prognosis, and targeted therapy.

**Supplementary Information:**

The online version contains supplementary material available at 10.1186/s12890-022-02179-5.

## Introduction

Lung cancer accounts for approximately one in ten (11.4%) confirmed cancer cases and one in five (18.0%) fatalities, according to Global Cancer Statistics 2020, with 2.2 million confirmed cases of cancer and 1.8 million deaths, ranking second and first, respectively, among all cancer types [1]. Non-small cell lung cancer (NSCLC), which includes adenocarcinoma of the lung and squamous cell carcinoma, is the most common histologic subtype of lung cancer according to the World Health Organization. It accounts for approximately 85% of lung malignancies [2]. Despite tremendous advances in the treatment of NSCLC, patients with NSCLC have a dismal five-year survival rate owing to severe side effects and resistance to surgery, radiation, and chemotherapy [3]. Therefore, it is vital to identify new antitumor targets to build on existing treatments. Angiogenesis plays a significant role in the growth, metastasis, and development of various malignancies [4–6]. Classical tumor angiogenesis has shown that when the tumor diameter exceeds 1–2 mm, activation of vascular endothelial cells is required for neovascularization to obtain blood supply and allow continued growth [7]. However, Maniotis [8] first proposed vasculogenic mimicry (VM), a novel microcirculation pattern that can supply blood to tumors, in his study of human melanoma. VM is defined as a tubular structure of neoplastic cells that can contain blood cells through self-deformation and matrix remodeling. The unique structure of VM can cause distant metastasis in the early stage of tumor development and predict a poor prognosis [9].

Rho GTPases are members of the Ras superfamily and participate in various essential biological processes, including cell cycle progression, cytoskeletal reorganization, and malignant transformation [10]; they are an essential part of tumor development and progression [11]. Rho GTPase-activating protein 25 (ARHGAP25) belongs to the Rho GTPase-activating protein (RhoGAP) family, which promotes endogenous hydrolysis of GTP and is a negative regulator of Rho GTPases. Thuault et al. revealed for the first time that ARHGAP25 inhibits the invasion of alveolar rhabdomyosarcoma (ARMS) cells [12]. Previous studies have shown that ARHGAP25 overexpression significantly inhibits the growth of many neoplastic cells and suppresses their migration and invasion [13–16]. However, the association between ARHGAP25 and VM in NSCLC has not been studied. In this study, we analyzed the effect of ARHGAP25 on NSCLC cell motility, and its expression in NSCLC tissue in relation to VM, so as to explore whether ARHGAP25 is beneficial to the prognosis of patients.

Ras homolog family member A (RhoA) is an isoform of the Rho family of small-molecule G proteins, which are involved in regulating various life processes, including cytoskeleton assembly, cell adhesion, motility, cycle progression, cytokinesis, and gene transcription [17]. RhoA is highly expressed in a range of tumor tissues, closely correlated with tumor malignancy, and plays an invaluable role in neoplastic metastasis and development [18]. In this study, we examined RhoA expression in NSCLC, its link to VM and ARHGAP25, and its potential clinical applicability.

## Materials and methods

### Patients and tissue samples

We collected 130 archived paraffin-embedded NSCLC tissue specimens, without preoperative radiotherapy or chemotherapy, with postoperative immunohistochemically confirmed lung squamous cell carcinoma (LUSC) and lung adenocarcinoma (LUAD), and paracancerous tumor-free tissues (> 5 cm from the tumor edge) [19], from the First Affiliated Hospital of Bengbu Medical College from January 2013 to December 2016. All patients had complete clinical, pathological, and follow-up data, and the enrolled cases were followed until death or up to 2021. To record the patients’ postoperative survival status, patients were followed-up telephonically at six-month intervals. Patient death or the December 2021 endpoint was used to compute overall survival time (OS), while patient death, relapse, or the December 2021 endpoint was used to calculate disease-free survival (DFS). The Ethics Committee of Bengbu Medical College authorized all patients to provide signed informed consent (NO. 2020KY035). The study adhered to the ethical guidelines of the Declaration of Helsinki. The American Joint Committee on Cancer’s 8th edition staging system was used to perform tumor-node-metastasis (TNM) staging of NSCLC. Table 1 shows the clinicopathological characteristics of the patients.

**Table 1:** Patients characteristics

Patients characteristics	Frequency (n)	Percentage (%)
*Age (years)*		
< 60	57	43.8
≥ 60	73	56.2
*Gender*		
Female	34	26.2
Male	96	73.8
*Smoking*		
No	68	52.3
Yes	62	47.7
*Tumor size (cm)*		
≤ 3	50	38.5
> 3	80	61.5
*Gross Type*		
Central	83	63.8
Peripheral	47	36.2
*Histologic Type*		
LUSC	75	57.7
LUAD	55	42.3
*Grade*		
Well	16	12.3
Moderate	81	62.3
Poor	33	25.4
*LNM*		
No	58	44.6
Yes	72	55.4
*TNM stage*		
I	40	30.8
II	33	25.4
III	57	43.8

TNM, tumor-node-metastasis; LNM, lymph node metastasis; LUAD, lung adenocarcinoma; LUSC, lung squamous cell carcinoma

### Immunohistochemistry

The collected paraffin specimens were serially sectioned at 4-μm thickness, deparaffinized with xylene, and dehydrated with graded alcohols after baking for 2 h. Sections were then washed in distilled water and phosphate-buffered saline (PBS) for 3 min each, respectively, and this was repeated thrice. Tissue slices were submerged in sodium citrate buffer (pH 6.0), pressure-cooked, chilled to 20 °C for antigen retrieval, and rinsed in PBS. To inhibit endogenous peroxidase, we performed tissue antigen retrieval and dripped a 3% hydrogen peroxide solution across all sections, followed by incubation for 15 min at 37 °C. The samples were washed thrice in PBS (pH 7.2) for 3 min each. The sections were incubated overnight at 4 °C with a few drops of rabbit monoclonal anti-ARHGAP25 (1:400, ab192020, Abcam, USA), mouse monoclonal anti-RhoA (1:100, ab54835, Abcam), and anti-CD34 (1:200, ab762, Abcam) antibodies. The secondary antibody was then added in a dropwise manner and the sections were allowed to sit for 30 min at 37 °C. Thereafter, sections were soaked in DAB solution, counterstained with hematoxylin, washed with distilled water, dehydrated with graded alcohols, and sealed with neutral resin.

### Evaluation of immunostaining

**Fig. 1:**
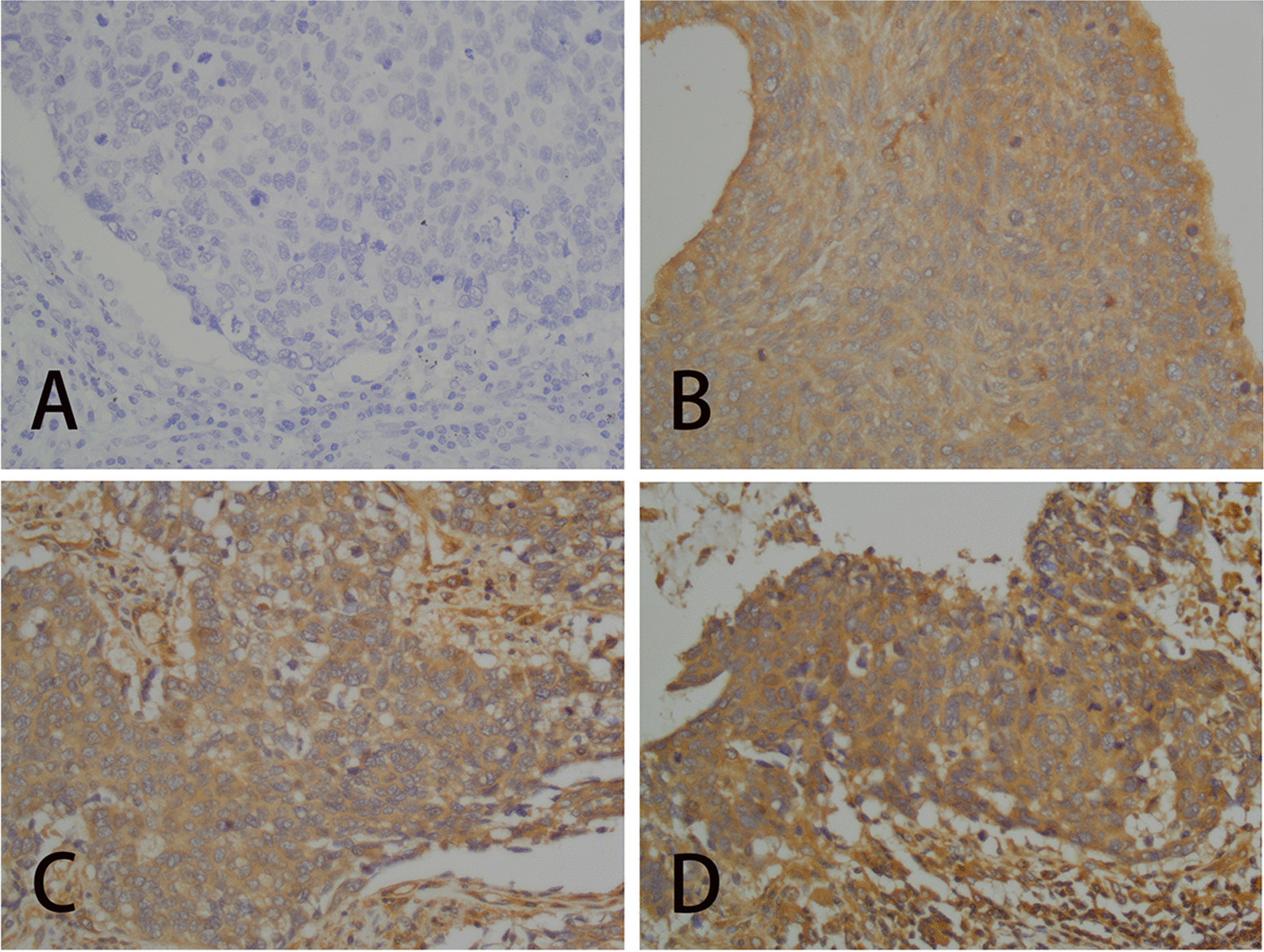
Staining intensity was assessed as an example of RhoA, cytoplasm positive (× 400). **A** no staining. **B** pale yellow staining. **C** tan staining. **D** brown staining

Two senior pathologists assessed the slides and performed immunohistochemical analysis. The immunohistochemical scores of ARHGAP25 and RhoA protein consisted of two parts: the staining intensity (0, no staining; 1, pale yellow staining; 2, tan staining; 3, brown staining) (Fig. 1)and the number of positive cells in the tumor tissue (0, < 10%; 1, 11–50%; 2, 51–75%; 3, > 75%), with the final score being the sum of the two. A three-point total score was deemed favorable, whereas a score below 3 was considered negative. We used the method of double staining with PAS-CD34 to show the structure of VM, and we considered that VM was present in this tumor when it appeared as CD34-negative but PAS-positive [20].

### Cell culture and SiRNA transfection

The Chinese Academy of Sciences Cell Bank (Shanghai, China) provided the lung cell lines. NCI-H1299 cells were cultivated in RPMI 1640 medium (Invitrogen, Carlsbad, CA, USA) containing 10% fetal bovine serum (FBS), whereas A549 cells were cultured in Dulbecco’s modified Eagle’s medium (DMEM) containing 10% FBS. The incubator temperature and CO_2_ content were set at 37 °C and 5%, respectively. Transfection of small interfering RNAs in NSCLC cells (Reebok Bio, Guangdong, China) according to the Lipofectamine 3000 reagent (Thermo Fisher Scientific) operating manual inhibited ARHGAP25 production when cell fusion rates reached 50–60% [21]. The knockdown efficiency of these siRNAs was assessed by RT-PCR and Western blotting.

### Quantitative reverse transcription-polymerase chain reaction analysis of ARHGAP25

Total RNA was extracted using the TRIzol technique. cDNA was prepared using the NovoStart SYBR qPCR SuperMix Plus (Novoprotein, China); quantitative reverse transcription-polymerase chain reaction (qRT-PCR) was used to measure the relative expression of *ARHGAP25* mRNA in cells [21, 22]. *β-actin* was used as an endogenous reference gene. The following primers were used: *ARHGAP25*: forward, 5'-GACAAGCGACTCTGATACAA-3', and reverse, 5'-GAAACATTTCCGGTTAGG-3'; *β-actin* forward, 5'-CATGTACGTTGCTATCCAGGC-3', and reverse, 5'-CTCCTTAATGTCACGCACGAT-3'.

### Western blotting of ARHGAP25

Radioimmunoprecipitation assay (RIPA) lysis buffer (Solarbio, China) was used to extract cellular proteins, and protein concentrations were determined using a bicinchoninic acid (BCA) protein quantification kit (Solarbio, China). Absorbance was recorded at 562 nm using a microplate reader. A complete protein (25 µg) was added to each well and proteins were separated using polyacrylamide gel electrophoresis (PAGE) with 8–12% sodium dodecyl sulfate (SDS) gels and transferred to polyvinylidene fluoride (PVDF) membranes [21, 22]. After blocking with 5% skim milk for 1 h, the membranes were incubated overnight at 4 °C with primary antibodies specific for ARHGAP25 (1: 1000, Abcam) and β-actin (1: 1000, Proteintech, China). Membranes were washed with tris-buffered saline with Tween® (TBST) on a shaker before adding the appropriate secondary antibodies (1:10,000, Proteintech, China). An enhanced chemiluminescence kit (Xinsaimei, China) was used to visualize immunoreactive protein bands.

### Detection of colony formation

Cells (1000 cells/well) were inoculated into six-well culture dishes and cultured in 2 ml DMEM (Gibco, USA) containing 10% FBS for two weeks. Thereafter, cells were fixed for 10 min in 1 mL of 4% neutral formaldehyde, washed three times with PBS, stained with 1 mL of 1% crystalline violet, photographed, and counted using ImageJ (ACTREC, Navi Mumbai, India) software [21].

### Cell migration assays

Cell migration ability was tested using a wound-healing experiment. A six-well plate was seeded with cells (5.0 × 10^5^ cells/well). When the cells were confluent, three vertical scratches were made in each well using a 100-μL sterile pipette tip. PBS was added slowly along the lateral wall, thereby flushing the cells shed on the wound. At 0 and 24 h, images were taken, samples were obtained, and the scratch lengths in each group were measured using ImageJ software [21].

### Staining of the actin cytoskeleton

Coomassie Brilliant Blue staining solution was used to examine the cytoskeleton of cultivated NSCLC cells. First, 8 × 10^4^ cells per group were seeded into six-well plates. The following day, adherent cells were fixed with 4% paraformaldehyde at room temperature for 20 min, infiltrated with 0.2% Triton-X-100 for 5 min, and stained with 1 mL Coomassie Brilliant Blue R-250 (0.2%). The cells were monitored and imaged using an inverted microscope after five rinses with distilled water [21].

### Statistical analysis

Statistical scores were obtained from our experimental data using SPSS 26.0 (IBM; Chicago, IL, USA). The correlations between the expression levels of ARHGAP25, RhoA, and VM, as well as clinicopathological data, were analyzed using the chi-square test. Spearman's correlation analysis was used to determine the relationship between ARHGAP25, RhoA, and VM. The association between each component and OS and DFS was compared using Kaplan–Meier analysis. Finally, Cox regression was used to estimate the risk factors that may influence patient prognosis using univariate and multifactor analyses.

## Results

### Relationship between ARHGAP25, RhoA, VM, and clinicopathological parameters

**Fig. 2:**
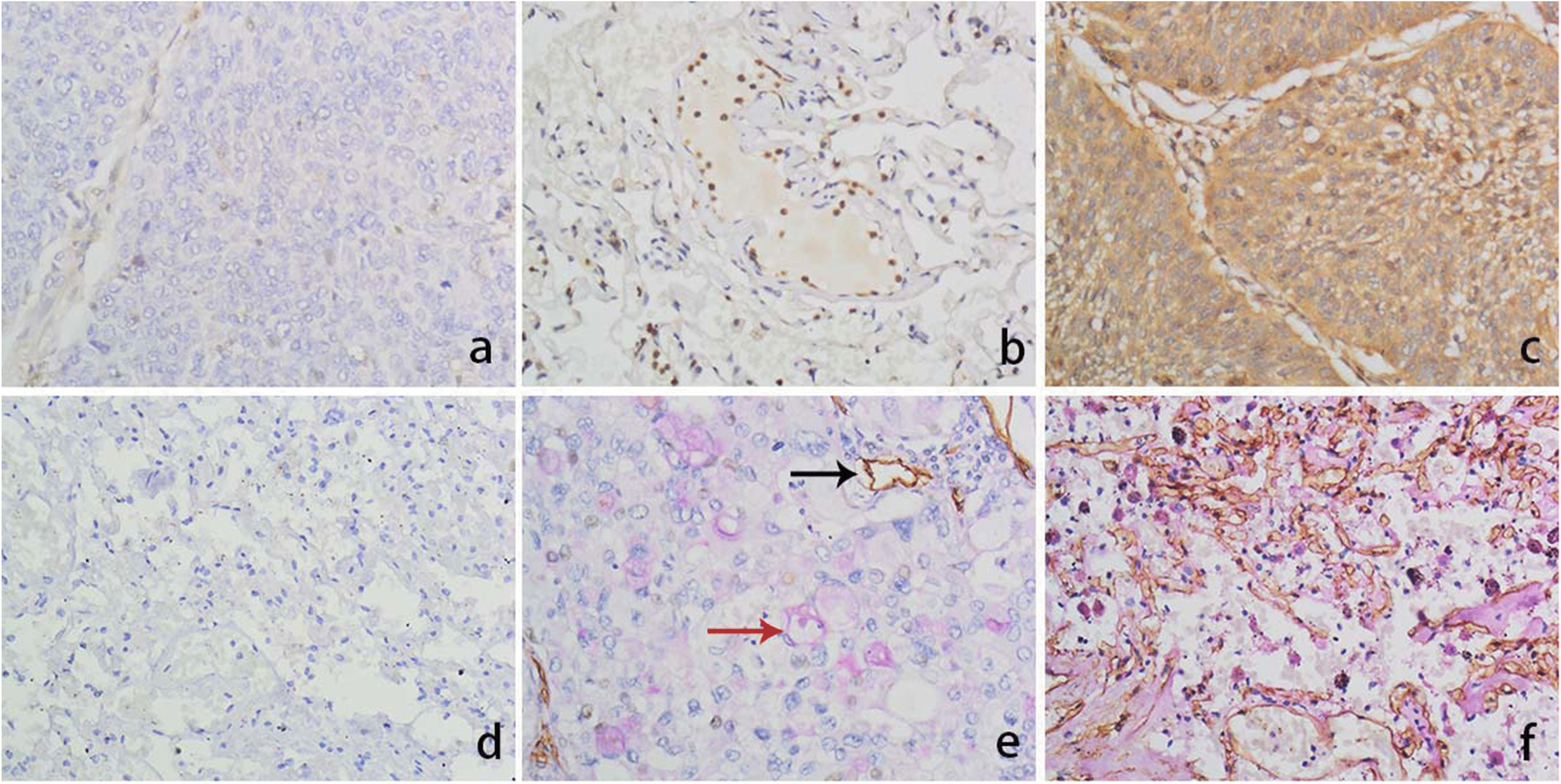
Immunohistochemical staining for ARHGAP25, RhoA and VM in NSCLC and normal paracancerous tissues. **a** ARHGAP25 staining is negative in NSCLC tissues (× 400). **b** ARHGAP25 positive staining in paracancerous tissues (× 400). **c** RhoA positive staining in NSCLC tissues (× 400). **d** RhoA staining is negative in paracancerous tissues (× 400). **e** In NSCLC tissues, VM staining is positive (× 400, red arrow is VM structure; black arrow is microvessel). **f** VM staining in paracancerous tissues is negative (× 400)

We used immunohistochemistry and SPSS 26.0 to count the positive rates of ARHGAP25, RhoA, and VM in NSCLC. ARHGAP25 was mainly localized in the nucleus, and ARHGAP25 levels were decreased in lung cancer tissues (48.5%, 63/130; Fig. 2a) compared to paracancerous tissues (63.1%, 82/130; Fig. 2b). The expression levels of ARHGAP25 in NSCLC tissues were significantly associated with tumor size (*P* = 0.015), TNM stage (*P* = 0.013), and lymph node metastasis (LNM; *P* = 0.015), but not with other clinicopathological factors (*P* > 0.05; Table 2). RhoA was primarily found in the cytoplasm, with a substantially higher expression rate in carcinomas (62.3%, 81/130; Fig. 2c) than in paracancerous tissues (18.5%, 24/130; Fig. 2d). The degree of RhoA expression was linked to tumor TNM stage (*P* = 0.002) and LNM (*P* = 0.001), but not to other clinicopathological factors (*P* > 0.05; Table 2). VM was present among the 130 NSCLC tissues collected in 48 cases (36.9%; Fig. 2e), whereas VM structures were absent in paracancerous tissues (Fig. 2f). The presence of VM structures in NSCLC was positively associated with larger tumor size (*P* = 0.016), poor grade (*P* = 0.004), TNM stage (*P* = 0.011), and LNM (*P* = 0.001); however, no other clinicopathological factors (*P* > 0.05) were found to be significantly related (Table 2).

**Table 2:** The correlation between ARHGAP25, or RhoA, or VM and clinicopathological characteristics in NSCLC tissues

Variables	ARHGAP25			RhoA			VM		
	Negative	Positive	P	Negative	Positive	P	Negative	Positive	P
Age (years)			0.566			0.588			0.474
< 60	31	26		20	37		34	23	
≥ 60	36	37		29	44		48	25	
Gender			0.314			0.246			0.521
Female	15	19		10	24		23	11	
Male	52	44		39	57		59	37	
Smoking			0.155			0.391			0.063
No	31	37		28	40		48	20	
Yes	36	26		21	41		34	28	
Tumor size (cm)			0.015			0.055			0.016
≤ 3	19	31		24	26		38	12	
> 3	48	32		25	55		44	36	
Gross type			0.417			0.518			0.533
Central	45	38		33	50		54	29	
Peripheral	22	25		16	31		28	19	
Histologic type			0.902			0.789			0.224
LUSC	39	36		29	46		44	31	
LUAD	28	27		20	35		38	17	
Grade			0.478			0.535			0.004
Well	8	8		4	12		16	0	
Moderate	39	42		32	49		46	35	
Poor	20	13		13	20		20	13	
LNM			0.015			0.001			0.001
No	23	35		31	27		46	12	
Yes	44	28		18	54		36	36	
TNM stage			0.013			0.002			0.011
I	13	27		23	17		31	9	
II	21	12		13	20		23	10	
III	33	24		13	44		28	29	

*NSCLC*, non-small cell lung cancer; *ARHGAP25*, Rho GTPase-activating protein 25; *RhoA*, Ras homolog family member A; *VM*, vasculogenic mimicry; *TNM*, tumor-node-metastasis; *LNM*, lymph node metastasis; *LUAD*, lung adenocarcinoma; *LUSC*, lung squamous cell carcinoma

### Relationship between ARHGAP25, RhoA, and VM

ARHGAP25 expression was negatively correlated with RhoA expression (r = -0.262, *P* = 0.003) and VM positivity (r = -0.232, *P* = 0.008), whereas RhoA expression was positively correlated with VM positivity (r = 0.365, *P* < 0.001; Table 3).

**Table 3:** Correlation among ARHGAP25 RhoA and VM in NSCLC

Variables	VM				RhoA			
	Negative	Positive	r	P	Negative	Positive	r	P
ARHGAP25			-0.232	0.008			− 0.262	0.003
Negative	35	32			17	50		
Positive	47	16			32	31		
VM							0.365	< 0.001
Negative					42	40		
Positive					7	41		

*NSCLC*, non-small cell lung cancer; *ARHGAP25*, Rho GTPase-activating protein 25; *RhoA*, Ras homolog family member A; *VM*, vasculogenic mimicry

### ARHGAP25 and RhoA expression levels, VM, and clinicopathological factors affect OS

**Fig. 3:**
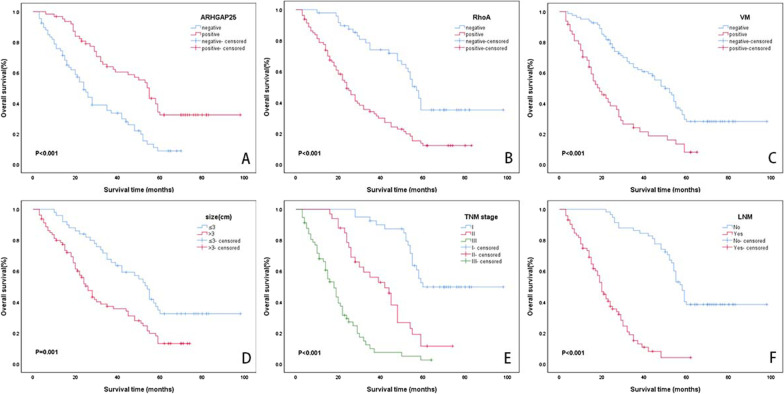
Kaplan–Meier analysis of OS in NSCLC patients. **A** Correlation of OS with ARHGAP25 (χ2 = 20.530, P < 0.001). **B** Correlation of OS with RhoA (χ2 = 21.604, P < 0.001). **C** Correlation of OS with VM (χ2 = 23.304, P < 0.001). **D** Correlation of OS with tumor size (χ2 = 12.072, P = 0.001). **E** Correlation of OS with TNM stage (χ2 = 83.749, P < 0.001). **F** Correlation of OS with LNM (χ2 = 74.429, P < 0.001)

We observed a median survival time of 35.0 months as well found a mean overall survival time of 44.6 ± 2.9 months, and the five-year OS rate was 20.9%, based on the Kaplan–Meier analysis of the five-year overall survival time of the collected NSCLC cases. The ARHGAP25-positive group (56.8 ± 4.1; 32.4%) displayed a considerably higher mean survival time and five-year OS rate than the ARHGAP25-negative samples (29.7 ± 2.6; 8.8%; χ2 = 20.530, *P* < 0.001; Fig. 3A). Similarly, the RhoA-positive group (31.9 ± 2.8; 12.1%) showed a significantly lower OS and OS rates than the RhoA-negative samples (62.1 ± 4.4; 35.2%; χ2 = 21.604, *P* < 0.001; Fig. 3B). The VM-positive samples (25.2 ± 2.9; 8.0%) displayed a significantly lower OS and OS rate than the VM-negative samples (53.9 ± 3.5; 28.1%; χ2 = 23.304, *P* < 0.001; Fig. 3C). OS and OS rates were reduced in patients with tumor size greater than 3 cm (32.9 ± 2.7 vs. 57.3 ± 4.5; 13.1% vs. 32.4%; χ2 = 12.072, *P* = 0.001; Fig. 3D). The OS and OS rates gradually decreased in patients with TNM stages I, II, and III (73.9 ± 4.1 vs 41.3 ± 3.1 vs. 20.2 ± 2.0; 49.7% vs. 11.4% vs. 2.5%; χ2 = 83.749, *P* < 0.001; Fig. 3E). Patients with LNM presented with a considerably lower five-year OS rate than those without LNM (22.6 ± 1.8 vs 66.5 ± 3.5; 4.0% vs. 38.4%; χ2 = 74.429, *P* < 0.001; Fig. 3F). Age, smoking, sex, gross tumor type, tumor grade, and histological type did not significantly affect OS (all *P* > 0.05).

### ARHGAP25 and RhoA expression levels, VM, and clinicopathological factors affect DFS

**Fig. 4:**
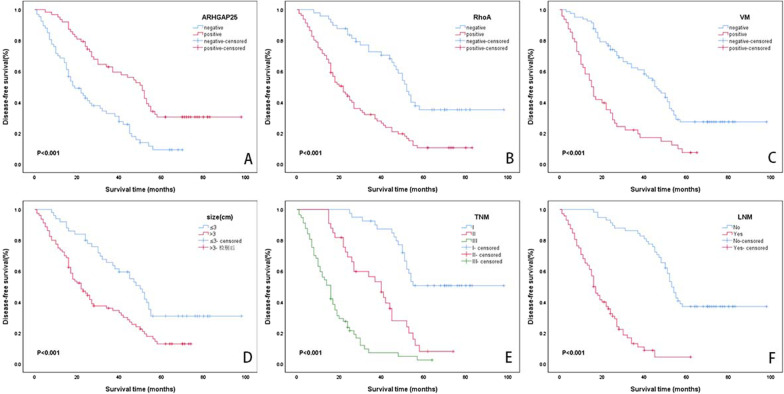
Kaplan–Meier analysis of DFS in NSCLC patients. **A** Correlation of DFS with ARHGAP25 (χ2 = 20.012, P < 0.001). **B** Correlation of DFS with RhoA (χ2 = 22.385, P < 0.001). **C** Correlation of DFS with VM (χ2 = 25.721, P < 0.001). **D** Correlation of DFS with tumor size (χ2 = 12.206, P < 0.001). **E** Correlation of DFS with TNM stage (χ2 = 83.827, P < 0.001). **F** Correlation of DFS with LNM (χ2 = 72.335, P < 0.001)

We observed a median survival time of 31.0 months and a mean disease-free survival time of 41.4 ± 2.9 months, and the five-year DFS rate was 19.9%, based on the Kaplan–Meier analysis of the five-year DFS time of the collected NSCLC cases. The DFS and DFS rates were significantly higher in ARHGAP25-positive samples (53.6 ± 4.1; 30.4%) than in ARHGAP25-negative samples (26.8 ± 2.6; 9.2%; χ2 = 20.012, *P* < 0.001; Fig. 4A). Similarly, the RhoA-positive group (28.8 ± 2.7; 10.5%) displayed significantly lower DFS and DFS rates than the RhoA-negative samples (59.3 ± 4.6; 35.1%; χ2 = 22.385, *P* < 0.001; Fig. 4B). The VM-positive samples (22.1 ± 2.8; 7.3%) had significantly lower DFS and DFS rates than the VM-negative samples (51.3 ± 3.6; 27.2%; χ2 = 25.721, *P* < 0.001; Fig. 4C). DFS and DFS rates were reduced in patients with tumor size greater than 3 cm (30.0 ± 2.6 vs. 54.5 ± 4.6; 12.9% vs. 30.8%; χ2 = 12.206, *P* < 0.001; Fig. 4D). The DFS and five-year DFS rates decreased in patients with TNM stages I, II, and III (72.2 ± 4.3 vs 38.2 ± 3.1 vs 17.6 ± 2.9; 50.6% vs. 7.9% vs. 2.4%; χ2 = 83.827, *P* < 0.001; Fig. 4E). Patients with LNM (20.2 ± 1.8; 4.3%) displayed considerably lower DFS and five-year DFS rates than those without LNM (63.6 ± 3.7; 37.1%; χ2 = 72.335, *P* < 0.001; Fig. 4F). Age, smoking, sex, general tumor type, tumor grade, and histological type did not significantly affect DFS (all *P* > 0.05).

### Cox regression analysis

Using Cox regression analysis, we identified the parameters that influence the prognosis in patients with NSCLC. Univariate analysis suggested that the expression levels of ARHGAP25 and RhoA, VM, tumor size, TNM stage, and LNM were strongly linked with OS and DFS, which are critical factors influencing the future prognosis with NSCLC. Multivariate analysis confirmed that ARHGAP25, RhoA expression, VM, tumor size, TNM stage, and LNM were closely associated with OS and DFS and may be employed as independent prognostic markers of NSCLC (Tables 4 and 5).

**Table 4:** Univariate and multivariate analysis of OS and clinicopathological variables

		Univariable analysis		Multivariable analysis	
Variables	Number	HR (95% CI)	P	HR (95% CI)	P
ARHGAP25		0.393(0.258–0.599)	< 0.001	0.408(0.262–0.636)	< 0.001
Negative	67				
Positive	63				
RHOA		2.747(1.754–4.300)	< 0.001	2.084(1.283–3.384)	0.003
Negative	49				
Positive	81				
VM		2.655(1.752–4.024)	< 0.001	1.872(1.195–2.933)	0.006
Negative	82				
Positive	48				
Tumor size (cm)		2.093(1.360–3.221)	0.001	2.187(1.327–3.605)	0.002
≤ 3	50				
> 3	80				
Age (years)		0.886(0.589–1.333)	0.561		
< 60	57				
≥ 60	73				
Gender		0.811(0.506–1.299)	0.383		
Female	34				
Male	96				
Smoking		1.221(0.815–1.832)	0.333		
No	68				
Yes	62				
Gross type		0.895(0.589–1.361)	0.604		
Central	83				
Peripheral	47				
Histologic type		1.342(0.889–2.025)	0.161		
LUSC	75				
LUAD	55				
Grade		1.345(0.953–1.898)	0.092		
Well	16				
Moderate	81				
Poor	33				
LNM		7.869(4.720–13.118)	< 0.001	4.534(2.379–8.643)	< 0.001
No	58				
Yes	72				
TNM stage		3.217(2.434–4.252)	< 0.001	2.338(1.655–3.303)	< 0.001
I	40				
II	33				
III	57				

NSCLC, non-small cell lung cancer; ARHGAP25, Rho GTPase-activating protein 25; RhoA, Ras homolog family member A; VM, vasculogenic mimicry; TNM, tumor-node-metastasis; LNM, lymph node metastasis; LUAD, lung adenocarcinoma; LUSC, lung squamous cell carcinoma

**Table 5:** Univariate and multivariate analysis of DFS and clinicopathological variables

		Univariable Analysis		Multivariable Analysis	
Variables	Number	HR (95% CI)	P	HR (95% CI)	P
ARHGAP25		0.405(0.269–0.612)	< 0.001	0.431(0.280–0.663)	< 0.001
Negative	67				
Positive	63				
RHOA		2.751(1.772–4.271)	< 0.001	2.031(1.263–3.266)	0.003
Negative	49				
Positive	81				
VM		2.722(1.814–4.084)	< 0.001	1.958(1.263–3.036)	0.003
Negative	82				
Positive	48				
Tumor size (cm)		2.078(1.361–3.172)	0.001	1.965(1.213–3.181)	0.006
≤ 3	50				
> 3	80				
Age (years)		0.923(0.618–1.378)	0.695		
< 60	57				
≥ 60	73				
Gender		0.878(0.557–1.385)	0.575		
Female	34				
Male	96				
Smoking		1.231(0.828–1.831)	0.305		
No	68				
Yes	62				
Gross type		0.834(0.551–1.262)	0.390		
Central	83				
Peripheral	47				
Histologic type		1.293(0.865–1.933)	0.211		
LUSC	75				
LUAD	55				
Grade		1.344(0.960–1.882)	0.085		
Well	16				
Moderate	81				
Poor	33				
LNM		7.361(4.464–12.137)	< 0.001	3.805(2.024–7.153)	< 0.001
No	58				
Yes	72				
TNM stage		3.172(2.411–4.172)	< 0.001	2.411(1.712–3.394)	< 0.001
I	40				
II	33				
III	57				

*NSCLC*, non-small cell lung cancer; *ARHGAP25*, Rho GTPase-activating protein 25; *RhoA*, Ras homolog family member A; *VM*, vasculogenic mimicry; *TNM*, tumor-node-metastasis; *LNM*, lymph node metastasis; *LUAD*, lung adenocarcinoma; *LUSC*, lung squamous cell carcinoma

### ARHGAP25 is downregulated in NSCLC cell lines

**Fig. 5:**
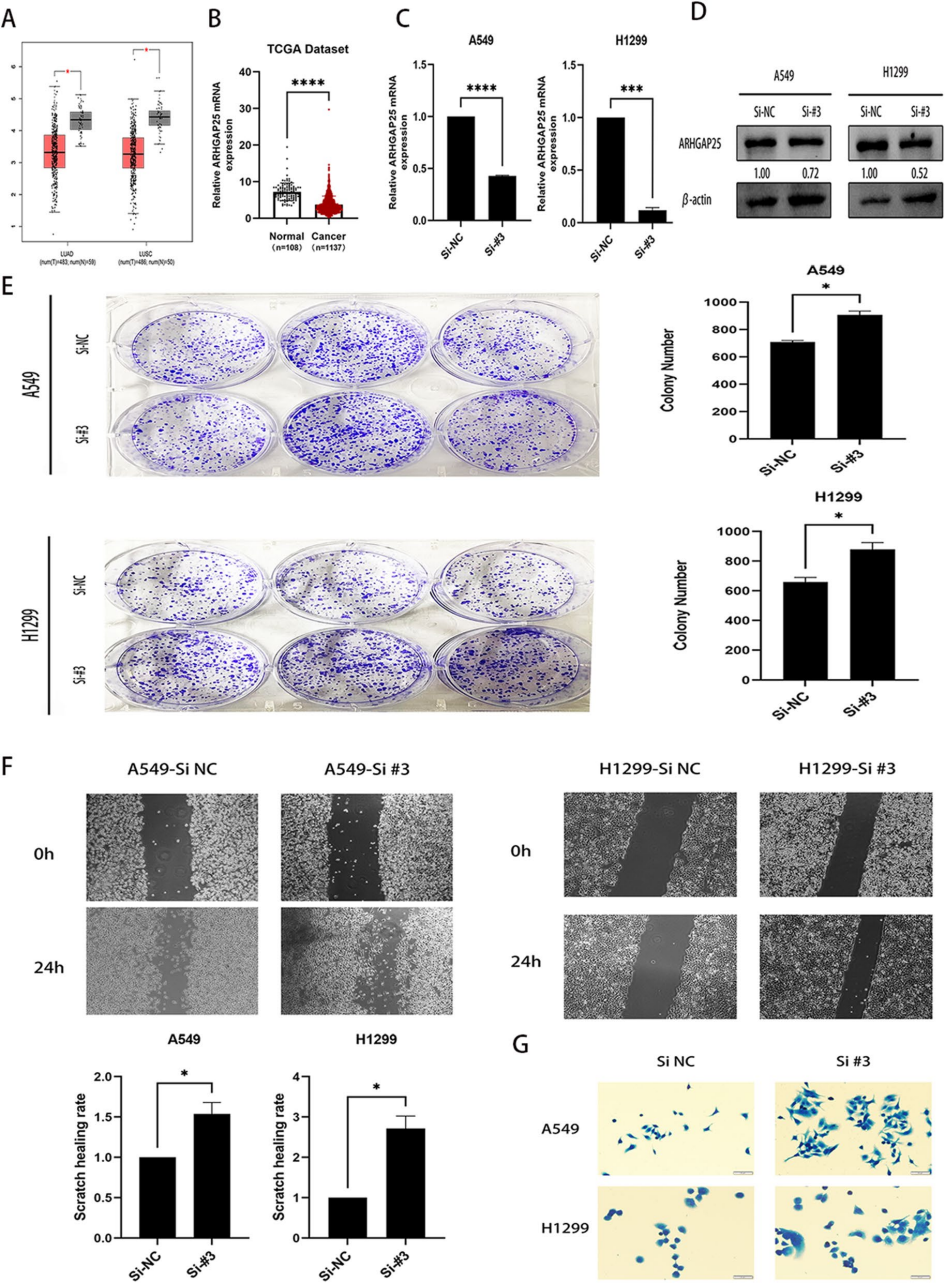
**A** Expression levels of ARHGAP25 gene in LUAD and LUSC were lower than those in normal tissues; red boxes, tumor tissues; gray boxes, normal tissues. **B** Expression levels of ARHGAP25 in NSCLC tissues were lower than those in normal. **C** The mRNA level of ARHGAP25 was considerably lower in the knockdown group. **D** The protein level of ARHGAP25 was considerably reduced in the knockdown group. **E** Effect of altering the expression of ARHGAP25 on the ability of cell clone formation. **F** Effect of altering the expression of ARHGAP25 on the ability of cell migration. **G** Effect of altered ARHGAP25 expression on cytoskeleton. *P < 0.05, **P < 0.01, ***P < 0.001, ****P < 0.0001. *Abbreviations*: Si NC, Control group; Si #3, knock-down group

The Cancer Genome Atlas (TCGA) database analysis (GEPIA) tool (http://gepia.cancer-pku.cn/) revealed that the level of ARHGAP25 was significantly lower in NSCLC (including 486 squamous lung carcinomas and 483 lung adenocarcinomas) tissues than in normal tissues (*P* < 0.001; Fig. 5A). To screen for gene expression profiles in NSCLC, we selected a qualified gene expression microarray dataset (TCGA) and determined that the expression level of ARHGAP25 was substantially reduced in NSCLC tissues (n = 1137) compared to normal tissues (n = 108) (Fig. 5B).

### Downregulation of ARHGAP25 enhances NSCLC cell proliferation and migration

We first knocked down *ARHGAP25* and used qRT-PCR and western blotting to confirm the results of siRNA knockdown in two NSCLC cell lines (A549 cells and H1299 cells). *ARHGAP25* mRNA and protein levels were considerably lower in the knockdown cases than in the negative control without siRNA knockdown (Fig. 5C and D). The original images of the Western blotting can be found in Additional files 1, 2, 3, 4. These results indicated that the *ARHGAP25* knockdown was successful. Subsequently, cell colony formation (Fig. 5E) and cell scratching assays (Fig. 5F) showed that lung cancer cells produced a higher number of colonies and a remarkable increase in the number of migrated cells after *ARHGAP25* expression was reduced. The results of actin cytoskeleton staining supported the enhanced cytoskeleton and increased motility observed in the knockdown group (Fig. 5G). In summary, the ability of A549 and H1299 cell lines to proliferate and migrate was substantially enhanced when *ARHGAP25* expression was reduced.

## Discussion

Depending on the TNM stage, histology, genetic modifications, and disease progression, treatments for NSCLC typically involve surgery, radiotherapy, chemotherapy, immunotherapy, and targeted molecular therapy, which can be administered alone or in combination [23]. For patients with early-stage NSCLC (stage I, II, and IIIA [N2 lymph node involvement usually found during surgery]), surgical resection is recommended. For stage II-IIIA disease, platinum-based adjuvant chemotherapy is recommended, with a 5.4% lower risk of death at five years, but with a high recurrence rate and relatively high toxicity [24]. Therefore, this study investigated the expression levels of ARHGAP25 and RHOA in NSCLC, as well as their relationship with VM, hoping to find more accurate and effective immune and molecular targeted therapies.

ARHGAP25 is localized on human chromosome 2p13 and is involved in the regulation of Rho family GTPases [25]. Rho GTPases are involved in cytoskeletal dynamics, cell cycle progression, transcriptional control, cell survival, and vesicle transport, all of which can influence cancer growth [10]. Some Rho GTPases promote cell cycle progression and gene transcription, which may explain their carcinogenic features, such as their ability to facilitate Ras-induced transformation [26]. Angiogenesis must be induced for a tumor to grow beyond a certain size and for malignant cells to release substances that encourage angiogenesis of neighboring pre-existing blood vessels. To promote neovascularization, certain Rho GTPases regulate the release of pro-angiogenic molecules [26]. RhoA is one of the most characteristic and universally highly expressed types of 20 Rho GTPs [27]. Rho GTPases have been demonstrated to influence diseased cell invasion and metastasis by controlling cytoskeletal contraction and cell membrane protrusion [28]. Negative regulators of Rac/Rho-like GTPases, GTPase-activating proteins (GAPs), reduce Rho GTPase activity by boosting the hydrolytic ability of Rho GTPases to convert activated GTP-binding status to inactivated GDP-binding status [29, 30].

ARHGAP25 is a Rac-specific GAP that is primarily expressed in hematopoietic cells. The invasion capability of alveolar rhabdomyosarcoma cells is controlled by the RhoE/ROCK/ARHGAP25 signaling pathway [12]. Furthermore, researchers discovered that ARHGAP25 was downregulated in colorectal cancer (CRC) and that upregulating ARHGAP25 decreased CRC metastasis both in vivo and externally [16]. One study revealed that the abnormal expression of ARHGAP25 reduces lung cancer cell proliferation and migration [14]. ARHGAP25 expression was found to be lower in NSCLC tissues (48.5%) than in nearby normal tissues (63.1%), and was correlated with larger tumor size (*P* = 0.015), LNM (*P* = 0.015), and later clinical stage (*P* = 0.013) in this study. Based on data from the TCGA database, we found that the expression level of ARHGAP25 was decreased in NSCLC compared to normal tissues, which is consistent with immunohistochemical results. Cell colony formation and wound healing assays, as well as actin cytoskeleton staining assays, supported that proliferation and migration were significantly enhanced in ARHGAP25 knockdown NSCLC cells. According to Kaplan–Meier analysis and Cox regression analyses, patients with positive ARHGAP25 expression displayed longer OS than those with negative ARHGAP25 expression. Previous studies have found that ARHGAP25 expression is an independent predictor of NSCLC prognosis. These findings imply that ARHGAP25 may function as a tumor suppressor, thereby slowing tumor growth.

RhoA is a small guanosine triphosphatase (GTPase) that belongs to the Ras superfamily and functions in cytoskeletal reorganization [31]. RhoA activity is controlled by Rho-associated coiled-coil protein kinase (ROCK), which phosphorylates its target protein [32] and disrupts certain biological functions, such as cell migration, adhesion, proliferation, contraction, and death. The activation of RhoA has been proven to increase the invasion and metastasis of several malignancies [33–35]. ROCK has been implicated in the production of VMs in liver cancer cell lines in several investigations [36, 37], and ROCK activation may be important in tumor cell VM regulation. The process by which tumor cells generate highly patterned vascular channels by relocating the F-actin cytoskeleton is characterized as VM, suggesting that RhoA might play a role in progression [21, 38]. In this study, RhoA expression in NSCLC was examined using immunohistochemistry. RhoA positivity was found in 62.3% of NSCLC tissues and 18.5% of normal tissues. RhoA overexpression was associated with LNM (*P* = 0.001) and later TNM stage (*P* = 0.013), implying that RhoA is relevant in NSCLC development. RhoA overexpression was associated with shorter OS and DFS, according to Kaplan–Meier survival and Cox regression analyses. Notably, RhoA expression and its impact on prognosis are comparable in NSCLC and other cancers. The expression level of RhoA was positively correlated with VM positivity and negatively correlated with ARHGAP25, according to the correlation analysis, which is consistent with earlier findings.

Differently from the classical model of tumor angiogenesis, VM formation is not dependent on endothelial cells [7]. Tumor cells mimic normal endothelial cells and form tubular VMs containing both erythrocytes and tumor cells. VM formation consists mainly of deformation of tumor cells, remodeling of the extracellular matrix, and vascular-like structures connected to existing blood vessels [39]. Studies have shown that the hypoxic microenvironment is closely associated with the development of VM, and that tumor cells form new blood vessels to obtain the required oxygen and nutrients [39]. Hypoxia promotes the differentiation of cancer stem cells to form endothelial-like structures, and epithelial-mesenchymal transition (EMT) promotes VM formation by reducing the expression of cell adhesion molecules, such as E-cadherin [40, 41]. Previously published studies showed 28.5%, 33.3%, 34%, 22.7%, and 19.2% positive rates of VM in gastric cancer, esophageal mesenchymal tumor, cutaneous melanoma, osteosarcoma, and colorectal cancer, respectively [42–46]. VM occurred in less than 50% of the cancer types studied. In this study, the VM positivity rate was 36.9%, which was linked to larger tumor size, poor differentiation, LNM, and late clinical stage, and the OS and DFS of VM-positive patients were also shorter. This demonstrates that tumor vascularization aided NSCLC invasion and metastasis, resulting in a poor prognosis, which was in line with previous research findings.

In this study, we analyzed the relationship between ARHGAP25, RhoA, and VM in NSCLC and initially explored the effects of ARHGAP25 and RhoA on vascularization, hoping to determine their value in predicting NSCLC metastasis, prognosis, and targeted therapy. Furthermore, through correlation analysis, we observed that VM positivity is negatively correlated with the expression of ARHGAP25, as opposed to RhoA. However, the specific mechanism of ARHGAP25 in VM remains unclear. The RhoA/ROCK signaling pathway has been implicated in the creation of VM in hepatocellular carcinoma, melanoma, carcinoma, and osteosarcoma [36, 38, 47, 48]; therefore, we inferred that RhoA has the same mechanism in NSCLC and that ARHGAP25, as a regulatory protein for RhoA, should also promote VM formation through negative regulation of RhoA. Due to the limitations of the conditions and the relatively simple experimental methods, the specific mechanism of ARHGAP25 and RhoA in promoting VM formation is still unclear and requires further study in future work.

## Conclusions

In conclusion, we showed that low ARHGAP25 expression and high RhoA expression are associated with VM and poor prognosis in patients with NSCLC. We believe that ARHGAP25 and RhoA may be used as novel prognostic biomarkers and therapeutic targets.

## Supplementary Information

**Additional file 1.** A549-H1299-actin-fields.

**Additional file 2.** A549-H1299-actin-strips.

**Additional file 3.** A549-H1299-ARHGAP25-fields.

**Additional file 4.** A549-H1299-ARHGAP25-strips.

## Data Availability

All data are available from the corresponding author upon reasonable request.

## Abbreviations

NSCLC: Non-small cell lung cancer
ARHGAP25: Rho GTPase-activating protein 25
RhoA: Ras homolog family member A
VM: Vasculogenic mimicry
TNM: Tumor-node-metastasis
LNM: Lymph node metastasis
LUAD: Lung adenocarcinoma
LUSC: Lung squamous cell carcinoma
OS: Overall survival
DFS: Disease-free survival
