# Supplementary figures and images for: Relationship between the expression of ARHGAP25 and RhoA in non-small cell lung cancer and vasculogenic mimicry
Source: BMC Pulm Med. 2022 Oct 7;22:377. doi: 10.1186/s12890-022-02179-5 (PMC9547444; doi:10.1186/s12890-022-02179-5)

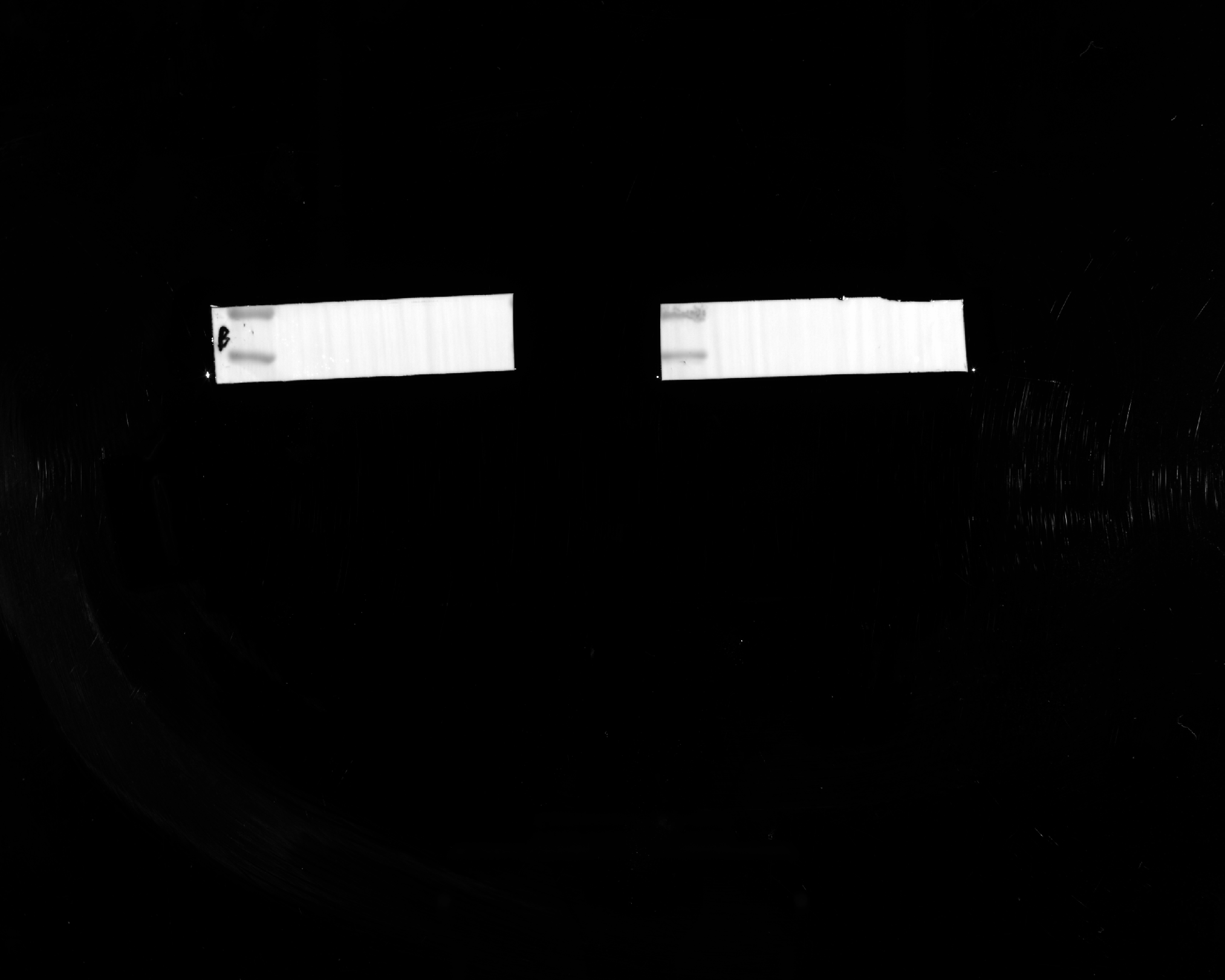

Supplement: Supplementary file 1 — Additional file 1. A549-H1299-actin-fields. [file 12890_2022_2179_MOESM1_ESM.tif]

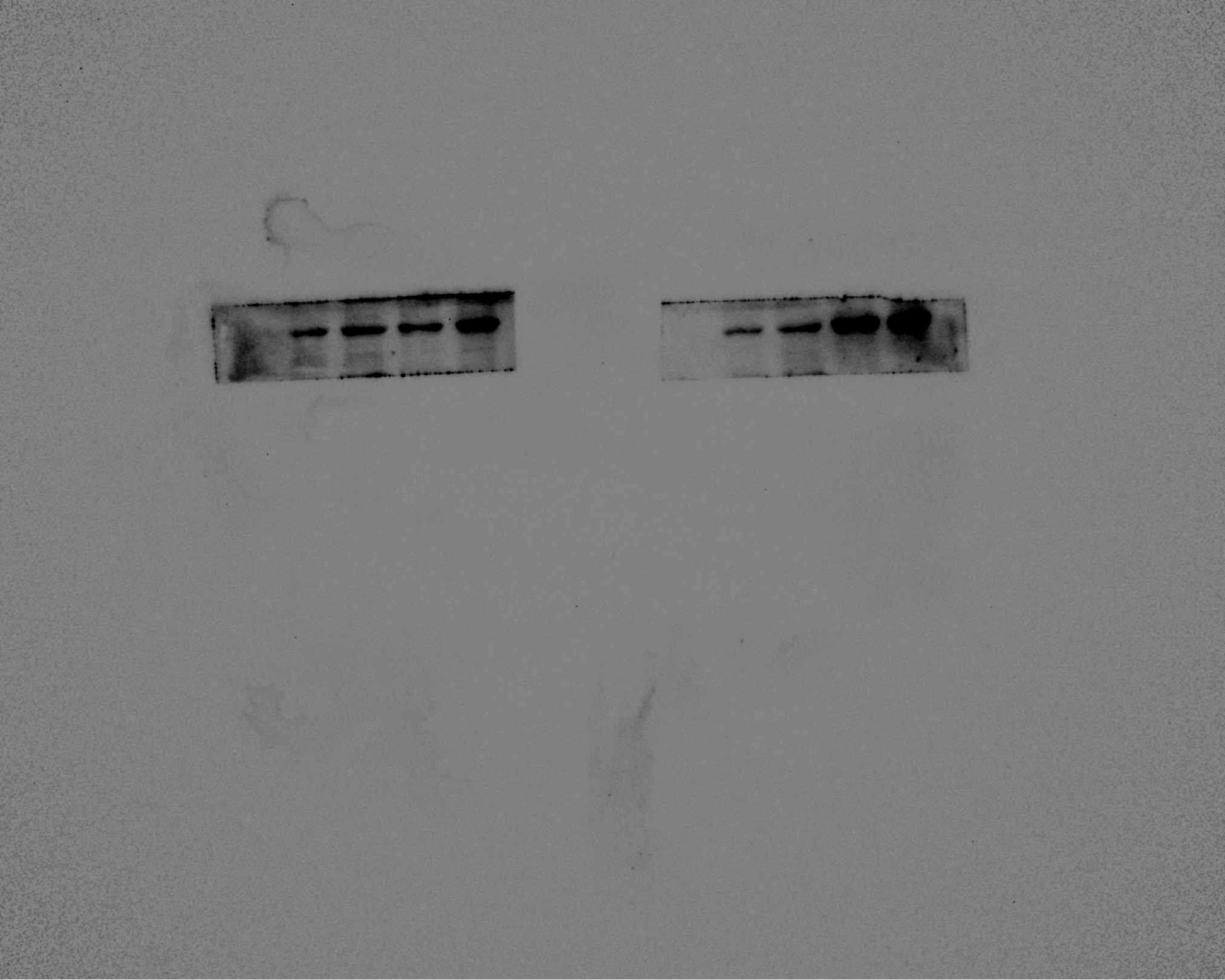

Supplement: Supplementary file 2 — Additional file 2. A549-H1299-actin-strips. [file 12890_2022_2179_MOESM2_ESM.tif]

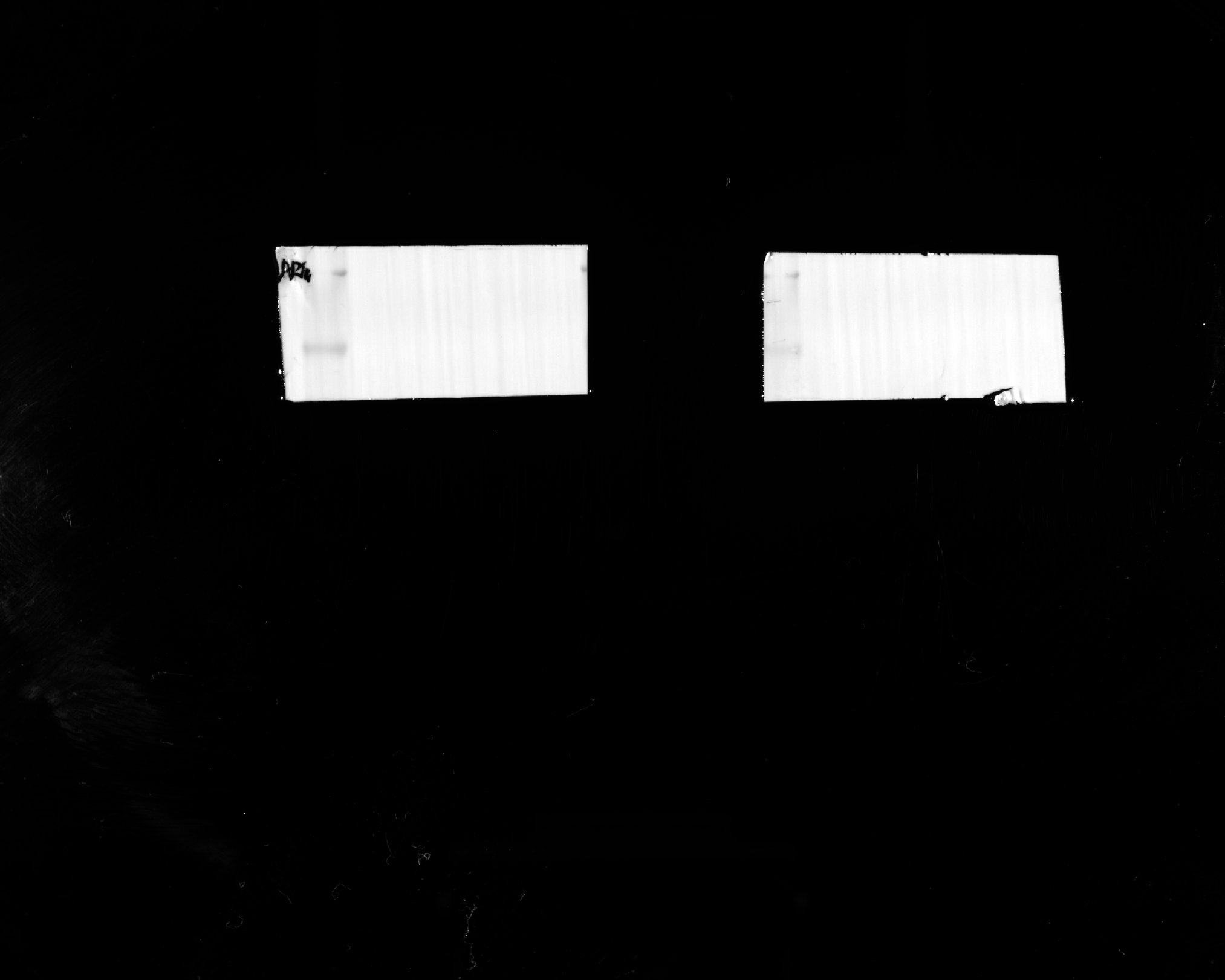

Supplement: Supplementary file 3 — Additional file 3. A549-H1299-ARHGAP25-fields. [file 12890_2022_2179_MOESM3_ESM.tif]

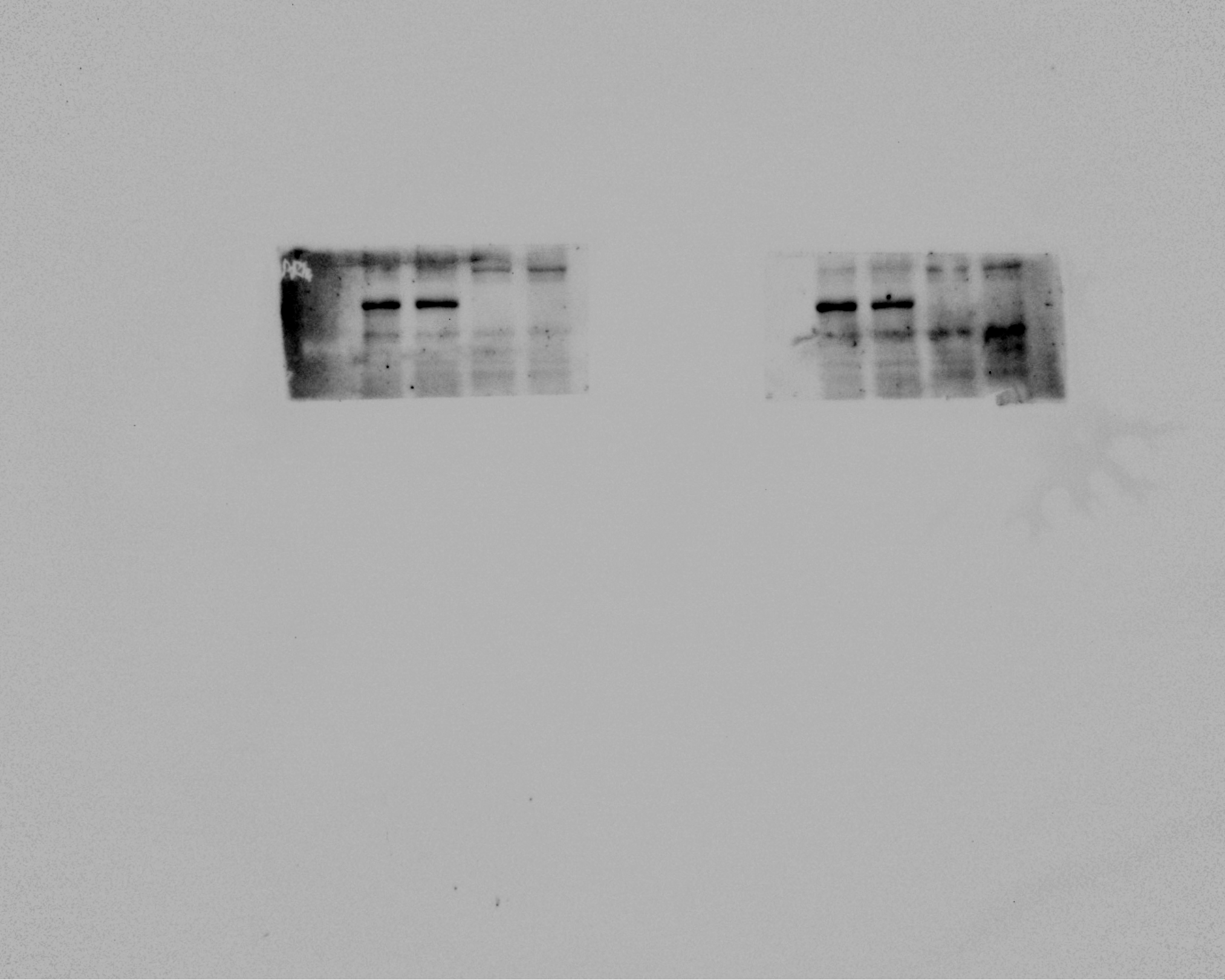

Supplement: Supplementary file 4 — Additional file 4. A549-H1299-ARHGAP25-strips. [file 12890_2022_2179_MOESM4_ESM.tif]
